# Reinforcement Techniques in Arthroscopic Repair of Large-to-Massive Rotator Cuff Tears: A Comparative Study of Superior Capsule Reconstruction and Patch Graft Augmentation

**DOI:** 10.3390/jcm13082276

**Published:** 2024-04-14

**Authors:** Jae-Sung Yee, Jin-Kwan Choi, Ki-Tae Kim, Ho-Won Lee, Yong-Beom Lee

**Affiliations:** 1 Division of Physical Examination, Gyeonggi Bukbu Regional Office of Military Manpower, Uijeongbu 11642, Republic of Korea; osjsyee@gmail.com; 2 Department of Orthopaedic Surgery, Hallym University Sacred Heart Hospital, Hallym University College of Medicine, Anyang 14068, Republic of Korea; omydamy@naver.com (J.-K.C.); kimkt8399@naver.com (K.-T.K.); 3 Department of Orthopaedic Surgery, Kangnam Sacred Heart Hospital, Hallym University College of Medicine, Seoul 07441, Republic of Korea; ehw80@hallym.or.kr

**Keywords:** rotator cuff tears, superior capsule reconstruction, patch graft augmentation, reinforcement, arthroscopic rotator cuff repair

## Abstract

**Background**: Large-to-massive rotator cuff tears (LMRCTs) present challenges in achieving successful repair due to factors such as muscle atrophy and tendon retraction. Arthroscopic rotator cuff repair (ARCR) with reinforcement techniques like superior capsule reconstruction (SCR) or patch graft augmentation (PGA) has emerged as a less invasive option to improve shoulder joint stability and prevent retear. This study aimed to compare the clinical and radiological outcomes of SCR and PGA as reinforcement techniques for the arthroscopic repair of LMRCTs. **Methods**: A single-center retrospective study was conducted on patients undergoing LMRCT repair between January 2019 and December 2021. Patients were divided into two groups: those receiving SCR (Group 1) and those receiving PGA (Group 2). Various clinical parameters including range of motion, functional scores, and radiological assessments were evaluated preoperatively and six months postoperatively. **Results**: Both SCR and PGA techniques demonstrated significant improvements in the range of motion and clinical scores postoperatively. However, Group 2 showed higher postoperative SST and UCLA scores compared to Group 1. Radiologically, there was a slightly higher retear rate in Group 2, although this was not statistically significant. Group 2 also had a shorter mean duration of surgery compared to Group 1. **Conclusions**: In the arthroscopic repair of LMRCTs, both SCR and PGA techniques exhibit favorable clinical and radiological outcomes. Despite the simplicity of PGA compared to SCR, it offers comparable results with a shorter surgical duration, making it a feasible reinforcement option for surgeons.

## 1. Introduction

Rotator cuff tears represent a common and challenging condition in orthopedic practice, with large-to-massive rotator cuff tears (LMRCTs) posing particular difficulties in management. These tears are associated with significant functional impairment, chronic pain, and a diminished quality of life for affected patients [1]. With an aging population and increasing recognition of the impact of shoulder disorders on daily activities and work performance, the prevalence and clinical significance of LMRCTs continues to grow.

Surgical intervention has emerged as a cornerstone in the management of LMRCTs, aiming to restore shoulder function, alleviate pain, and improve overall patient outcomes. Non-operative approaches, while beneficial for certain patients, often fall short in addressing the underlying pathology of extensive rotator cuff tears [2]. Consequently, there is a growing emphasis on the development and refinement of surgical techniques to optimize outcomes for patients with LMRCTs.

Recent advancements in surgical management have introduced novel approaches and techniques that hold promise for improving patient outcomes. Arthroscopic rotator cuff repair (ARCR) has gained popularity due to its minimally invasive nature and potential for reduced postoperative morbidity. It is essential to reduce the footprints of rotator cuff tears and repair them firmly. However, for LMRCTs, repairing without causing residual defects and preventing the retear of the repaired LMRCTs is difficult for several reasons, such as muscle atrophy, fatty infiltration, and tendon retraction with inelasticity [1,2].

In these cases, we can consider ARCR coupled with superior capsule reconstruction (SCR) [3] or patch graft augmentation (PGA) [4,5,6,7] reinforcement. Both operative techniques are less invasive than shoulder arthroplasty and improve shoulder joint stability, especially the superior stability of the glenohumeral joint. This helps prevent the superior migration of the humeral head, which aggravates arthritis around the shoulder joints and thereby requires patients to undergo shoulder arthroplasty. In addition, these techniques reduce the incidence of retears, improve clinical outcomes, and are viable options for complicated cases in which a significant failure rate is anticipated [8].

SCR was first introduced by Mihata et al. [3]. It achieves functional improvement by achieving glenohumeral joint stability. According to a recent study [9], SCR reinforcement before ARCR has been shown to improve cuff integrity not only for irreparable LMRCTs but also for reparable LMRCTs. In contrast, PGA involves covering the defect by augmenting it with a graft. Recently, PGA using allogeneic dermal patches has been widely performed, with successful outcomes reported in many studies [5,10].

Recent advancements and controversies in the management of LMRCTs continue to shape clinical practice and drive research efforts. Since there has been no research on reinforcement options for the arthroscopic repair of LMRCTs, this study aimed to compare the clinical and radiological outcomes of SCR and PGA as reinforcement techniques for the arthroscopic repair of LMRCTs. We hypothesized that the PGA technique would be comparable to the SCR technique for ARCR.

## 2. Materials and Methods

### 2.1. Patient Enrollment

**Figure 1:**
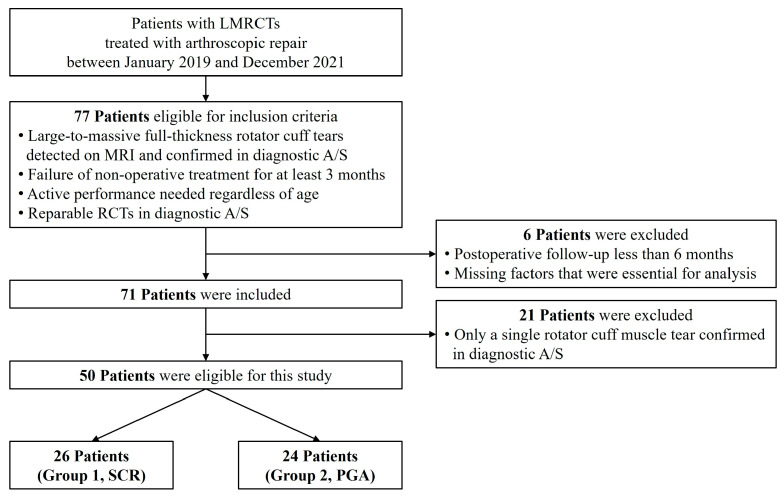
Patient flowchart. LMRCTs, large-to-massive rotator cuff tears; MRI, magnetic resonance imaging; A/S, arthroscopy; SCR, superior capsule reconstruction; PGA, patch graft augmentation.

**Figure 2:**
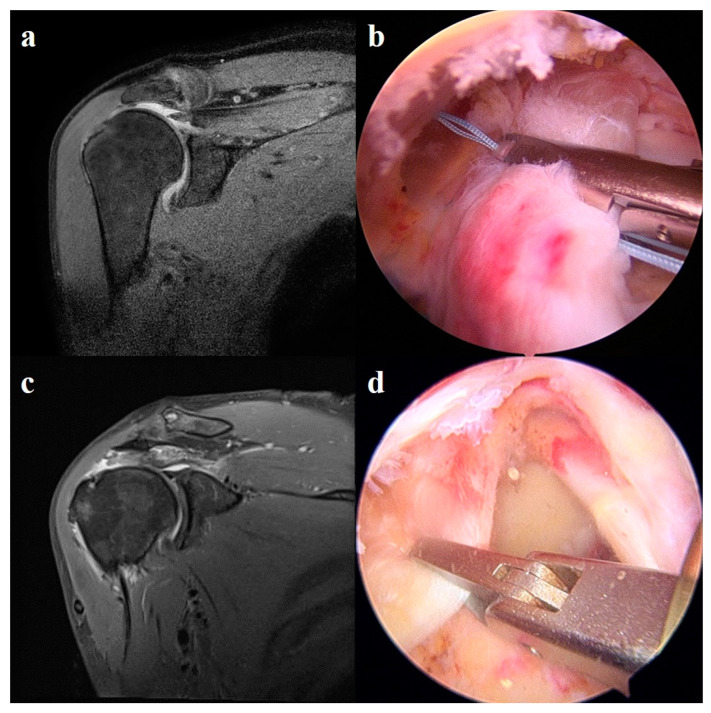
Preoperative MRI and A/S images. (**a**,**b**) LMRCTs are shown in a preoperative MRI image of a patient who underwent PGA reinforcement. Reparable LMRCTs were confirmed using A/S. (**c**,**d**) LMRCTs are shown in a preoperative MRI image of a patient who underwent SCR reinforcement. Reparable LMRCTs were confirmed using A/S. LMRCTs, large-to-massive rotator cuff tears; MRI, magnetic resonance imaging; A/S, arthroscopy; SCR, superior capsule reconstruction; PGA, patch graft augmentation.

This single-center retrospective study included patients who underwent LMRCTs and arthroscopic repair performed by a single surgeon between January 2019 and December 2021 (Figure 1). The inclusion criteria were as follows: (1) large-to-massive full-thickness RCTs that were detected on preoperative magnetic resonance imaging (MRI) and confirmed using diagnostic arthroscopy, the (2) failure of nonoperative treatment for at least 3 months, (3) patients who needed active performance regardless of age, and (4) partially or completely reparable RCTs confirmed using diagnostic arthroscopy (Figure 2). The exclusion criteria for this study were: (1) patients who underwent a postoperative follow-up period of less than six months, (2) those whose items were not preoperatively or postoperatively evaluated, and (3) those who had only a single tear of any rotator cuff muscle confirmed arthroscopically. We defined patients who underwent SCR reinforcement as Group 1 and those who underwent PGA reinforcement as Group 2. This study was approved by the Hallym University Sacred Heart Hospital Institutional Review Board (IRB File No. HALLYM 2020-07-034-008). Because of the retrospective nature of the study, the necessity for obtaining informed consent was waived.

### 2.2. Range of Motion Evaluation

For all patients, active range of motion (ROM), including forward elevation (FE) and internal rotation (IR), was measured preoperatively and six months after surgery. FE was evaluated using a goniometer with the patients in a standing position. IR was evaluated by checking the highest level at the midline that the patients reached when stretching a “hitch-hiking” thumb behind themselves. For statistical analysis, we designated the sacrum as 0 points, and 1 point was added for each level above this [11]. Additionally, we subtracted preoperative IR points from postoperative IR points and mentioned it as “IR level difference”. All ROM measurements were performed by a clinical researcher blinded to the study.

### 2.3. Functional and Clinical Satisfaction Evaluation

To assess the functional and clinical satisfaction outcomes of the patients, the pain visual analog scale (P-VAS) and various clinical scores, including the Simple Shoulder Test score (SST), the University of California in Los Angeles shoulder score (UCLA), the American Shoulder and Elbow Surgeons score (ASES), and the Constant Shoulder Score (CSS), were evaluated preoperatively and six months after surgery. These scores were assessed by a clinical researcher who was blinded to this study.

### 2.4. Radiological Evaluation

All patients underwent preoperative MRI to evaluate the RCTs. According to Iannotti et al. [12], an MRI scan was performed on all patients six months after the operation to confirm retear. To reduce measurement errors, both the preoperative and postoperative MRI scans were evaluated by an orthopedic fellow. Grading was performed by dividing the patients into three groups: a group in which retear was not observed at all, a group in which partial-thickness retear was observed, and a group in which full-thickness retear was observed.

### 2.5. Surgical Technique

Before surgery, patients were informed about the reinforcement options (SGA and PGA) that could be used for the surgical procedure after ARCR, along with their respective advantages and disadvantages [3,4,5,6,7]. They were allowed to choose a reinforcement option.

#### 2.5.1. Preparation Procedure

All surgeries were performed under general anesthesia with the patient in the beach chair position. The arthroscopic normal saline pump pressure was set between 30 and 50 mmHg. After marking the anatomical landmarks, we made four incisions to create arthroscopic portals (posterior, lateral, accessory anterolateral, and anterior). Using these four portals, we assessed the patient’s shoulder to explore and treat intra-articular lesions, such as labral tears and bicep long-head tendon tears, and extra-articular lesions, such as pathologic bursal tissues that impeded the clearance of space and the subacromial bone spur. Tenodesis or tenotomy was performed when a partial or full tear or an absence of a bicep long-head tendon was observed. Additionally, capsulectomy was performed if capsulitis was present. Each RCT was debrided until a viable tissue margin appeared. Acromioplasty was performed for the subacromial bone spur using an arthroscopic electric burr. We also debrided the superior glenoid and rotator cuff footprints of the greater tuberosity to reveal bleeding from the cancellous bone.

#### 2.5.2. Superior Capsule Reconstruction

After preparation, we first repaired the subscapularis muscle using the SpeedBridge technique, if indicated. We then positioned the patient’s arm in a fully adducted and internally rotated position to determine the adequate size of the allogeneic dermal patch to be inserted within the resistible range of tension. After measuring the appropriate size, a four-stranded suture anchor was inserted on the anterior and posterior sides of the well-prepared superior edge of the glenoid, medial to the superior labrum. We chose two strands from each suture anchor and pulled them outside the patient’s shoulder through the accessory anterolateral portal. The other paired strands of each suture anchor were pulled out through the anterior portal for the anterior suture anchor and through the posterior portal for the posterior suture anchor.

**Figure 3:**
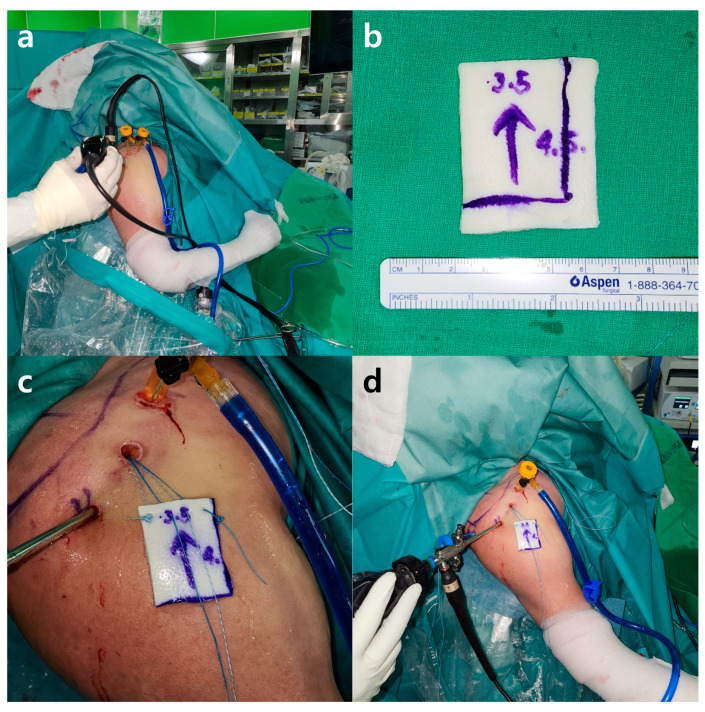
External images of the right shoulder that underwent SCR reinforcement before ARCR in the beach chair position. (**a**) The patient’s arm was placed in a fully adducted and internally rotated position. (**b**) An appropriately sized allogenic dermal patch. A lateral-to-medial arrow was marked to avoid confusion. (**c**) Using an EXPRESSEW^®^ Flexible Suture Passer, all four strands were passed to the medial side of the allogenic dermal patch. Mega knots, also known as Mulberry knots, were made with the most anterior and posterior strands. (**d**) The allogenic dermal patch was pulled into the patient’s shoulder through the accessory anterolateral portal incision site. SCR, superior capsule reconstruction; ARCR, arthroscopic rotator cuff repair.

Outside the patient’s shoulder, we passed all four strands on the medial side of the allogenic dermal patch we prepared, using an EXPRESSEW^®^ Flexible Suture Passer (DePuy-Mitek Inc., Raynham, MA, USA). We made a mega-knot with the anterior and posterior strands. We then pulled the paired strands that came out through the anterior and posterior portals to place an allogeneic dermal patch into the patient’s shoulder through the accessory anterolateral portal, as mentioned above. Special care was taken to avoid strand tangling during this process (Figure 3). After positioning the allogeneic dermal patch underneath the patient’s native rotator cuff tendon, the supraspinatus and infraspinatus muscles were augmented with strands that passed through the allogeneic dermal patch [13]. All ties were made using the Samsung Medical Center (SMC) knot technique [14].

**Figure 4:**
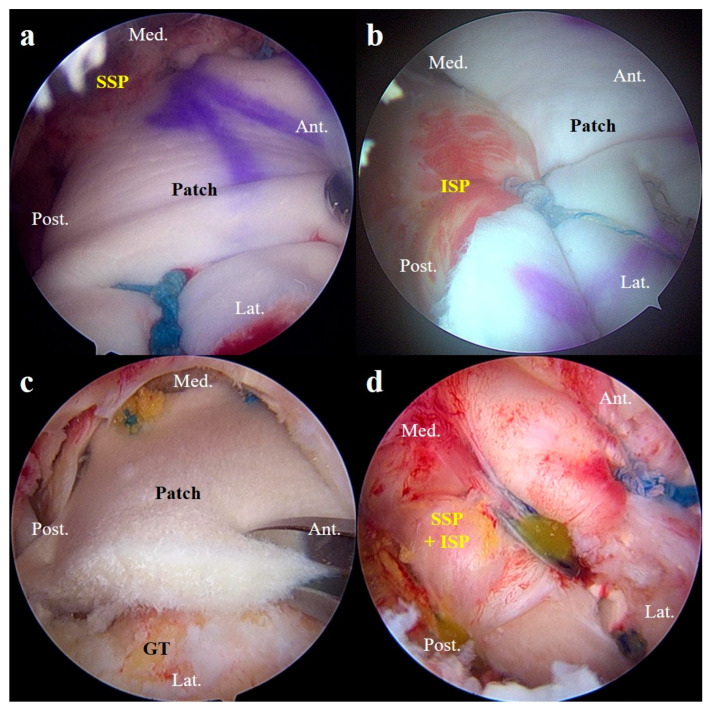
A/S images of the patients who underwent ARCR and SCR reinforcement. (**a**,**b**) This patient underwent SCR and was partially repaired with the remnants of the supraspinatus and infraspinatus muscles. (**c**,**d**) This other patient underwent SCR and the supraspinatus and infraspinatus muscles over the patch graft were completely repaired. SSP, supraspinatus muscle; ISP, infraspinatus muscle; GT, greater tuberosity; A/S, arthroscopy; ARCR, arthroscopic rotator cuff repair; SCR, superior capsule reconstruction; Ant., anterior; Post., posterior; Med., medial; Lat., lateral.

For lateral fixation, we inserted two suture anchors on the rotator cuff footprint of the greater tuberosity. We then used the SpeedBridge technique with a double tendon perforation per anchor [15] to fix the lateral side of the allogeneic dermal patch. Additional side-to-side sutures were brought from the infraspinatus to the graft itself to incorporate the infraspinatus tendon and graft into one soft tissue construct. Similarly, side-to-side stitches were placed anteriorly if there was any remaining anterior rotator interval tissue, but not in the subscapularis tissue. Once SCR was complete, tenodesis of the long head of the biceps tendon was performed to complete the procedure (Figure 4) [16].

#### 2.5.3. Patch Graft Augmentation

After preparation, if indicated, we started to repair the subscapularis muscle using the SpeedBridge technique and simultaneously performed tenodesis of the long head of the biceps tendon, if needed. We then reduced the remaining RCTs to their footprints as much as possible within tolerable tension to prevent retear and marked the appropriate points to insert suture anchors. We augmented the supraspinatus and infraspinatus muscles with strands from suture anchors using an EXPRESSEW^®^ Flexible Suture Passer. All simple ties were made using the SMC knot technique, except for the anterior and posterior sutures.

Following augmentation, the size of the defect was measured using an arthroscopic probe to prepare an adequately sized allogeneic dermal patch. After measuring the appropriate size, we pulled two strands from the anterior and posterior sutures and pulled them outside the patient’s shoulder through the accessory anterolateral portal. The other paired strands of each suture were pulled out through the anterior portal for the anterior sutures and through the posterior portals for the posterior sutures.

**Figure 5:**
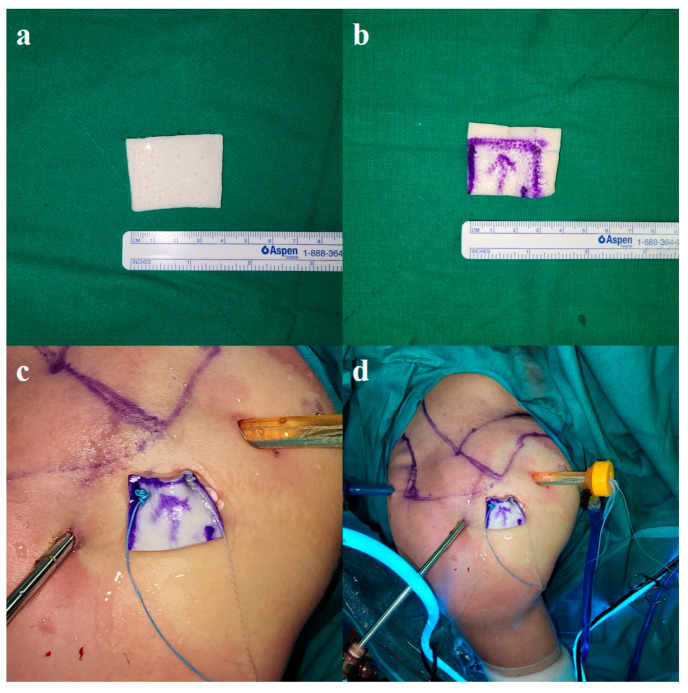
External images of the right shoulder that underwent PGA reinforcement after ARCR in the beach chair position. (**a**) BellaCell HD™ (Hans BioMed Corp., Seoul, Republic of Korea); (**b**) An appropriately sized allogenic dermal patch. A lateral-to-medial arrow was marked to avoid confusion. (**c**) Using an EXPRESSEW^®^ Flexible Suture Passer, two strands were passed to the medial side of the allogenic dermal patch. Mega knots were made anteriorly and posteriorly. (**d**) The allogenic dermal patch was pulled into the patient’s shoulder through the accessory anterolateral portal incision site. PGA, patch graft augmentation; ARCR, arthroscopic rotator cuff repair.

As previously mentioned, using an EXPRESSEW^®^ Flexible Suture Passer, we passed the two strands anteriorly and posteriorly on the medial side of the allogenic dermal patch we prepared. We created a mega-knot for each strand. We then pulled the paired strands that came out through the anterior and posterior portals to place the allogeneic dermal patch into the patient’s shoulder through the accessory anterolateral portal. Special care was taken to prevent the strand from getting tangled during this process (Figure 5).

**Figure 6:**
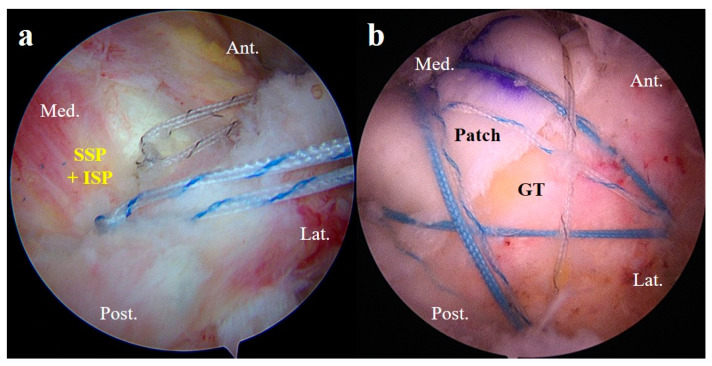
A/S images of the patients who underwent ARCR and PGA reinforcement. (**a**) The supraspinatus and infraspinatus muscles were repaired as much as possible using the suture-bridge technique. (**b**) Using the PGA technique, reinforcement was done over the defect to cover the greater tuberosity. SSP, supraspinatus muscle; ISP, infraspinatus muscle; GT, greater tuberosity; A/S, arthroscopy; ARCR, arthroscopic rotator cuff repair; PGA, patch graft augmentation; Ant., anterior; Post., posterior; Med., medial; Lat., lateral.

After positioning the allogeneic dermal patch on top of the native rotator cuff tendon, the suture-bridge technique was used to fix the lateral side of the allogeneic dermal patch. Two or three lateral anchors were inserted depending on the size of the defect (Figure 6) [13].

### 2.6. Postoperative Rehabilitation

To prevent immediate postoperative failure, a brace immobilizer with an abduction function was applied, and the operated shoulder was immobilized for three to four weeks. The rehabilitation protocol was started the day after surgery by initiating isometric rotator cuff exercises and the relaxation of the muscles around the shoulder girdle. After the immobilization period, the patients started passive and active-assisted exercises for FE and external rotation to avoid pain. After six weeks, strengthening exercises were initiated for the rotator cuff and the scapular stabilizers. Three months postoperatively, patients were allowed to practice light sports activities. A full return to heavy labor or sports was allowed after six months depending on each patient’s functional recovery [13].

### 2.7. Statistical Analysis

All statistical analyses were performed using SPSS software (version 27.0; IBM Corp., Armonk, NY, USA). The Chi-squared test was used to determine differences in nominal variables, and the Mann–Whitney U test was used to compare continuous variables between the two groups. The Mann–Whitney U test was used to compare the preoperative and postoperative results between Group 1 and 2. The preoperative and postoperative results included the ROM and clinical scores. Statistical significance was set at *p* < 0.05. Descriptive statistics are presented as mean ± standard deviation.

## 3. Results

### 3.1. Patient Characteristics

Of the 77 patients reviewed, 50 met the study inclusion criteria. As mentioned previously, these patients were divided into two groups. The patients who underwent SCR reinforcement were classified as Group 1, whereas those who underwent PGA reinforcement were classified as Group 2 (Group 1, 26 patients; Group 2, 24 patients). The two groups did not show statistically significant differences in their age, sex, the side of involvement, the duration of their symptoms, smoking, traumatic events, same-side operation history, and types of concomitant procedure. However, the mean duration of surgery differed significantly between the two groups (*p* = 0.021) (Table 1).

### 3.2. Range of Motion Outcomes

All the factors to compare the ranges of motion showed no statistically significant difference between the two groups, except the mean points of the preoperative IR. The mean points of the preoperative IR in Group 1 were less than those in Group 2 (*p* = 0.021) (Table 2).

### 3.3. Functional and Clinical Satisfaction Outcomes

Comparing the two groups, only the mean SSTs and UCLAs, which were evaluated postoperatively, showed statistically significant differences (SST, *p* = 0.034; UCLA, *p* = 0.001). Both scores were higher in Group 2 (Table 3).

### 3.4. Radiological Outcomes

**Figure 7:**
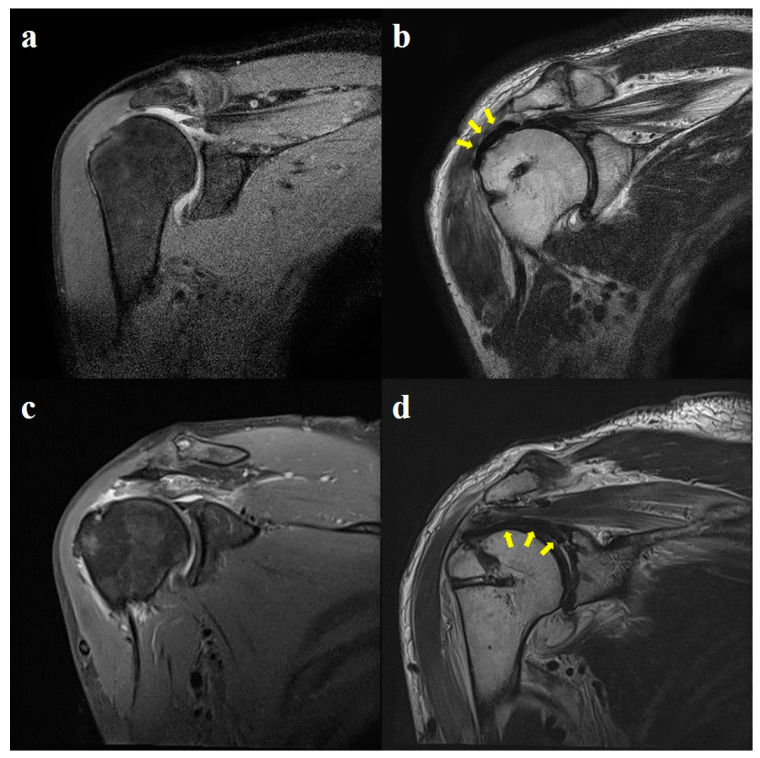
Preoperative and postoperative MRI images. (**a**,**b**) LMRCTs are shown in the preoperative MRI image. In the postoperative MRI image, LMRCTs have been repaired, and the patch (yellow arrows) fully covers the defect and the greater tuberosity using PGA reinforcement. (**c**,**d**) LMRCTs are shown in the preoperative MRI image. In the postoperative MRI image, SCR using a patch (yellow arrows) was performed, and this fully covered the greater tuberosity. LMRCTs were well repaired over the patch. LMRCTs, large-to-massive rotator cuff tears; MRI, magnetic resonance imaging; SCR, superior capsule reconstruction; PGA, patch graft augmentation.

Eighteen patients (69.2%) in Group 1 and sixteen patients (66.7%) in Group 2 had no radiological evidence of retear on MRI scans evaluated six months after surgery; however, this difference was not statistically significant between the two groups (*p* = 0.846). Eight patients (30.8%) in Group 1 had evidence of a retear. Of the eight patients, six had partial-thickness tears, and two had full-thickness tears. Eight patients (33.3%) in Group 2 had evidence of a retear, of which five had partial-thickness tears and three had full-thickness tears. The retear rate was higher in Group 2, but the difference between the two groups was not statistically significant (*p* = 0.590) (Table 4, Figure 7).

## 4. Discussion

Overall, the study aimed to compare the clinical and radiological outcomes of SCR and PGA as reinforcement techniques for the arthroscopic repair of LMRCTs. We hypothesized that the PGA technique would be comparable to the SCR technique for ARCR.

The study included 50 patients with LMRCTs who underwent either SCR or PGA reinforcement. Various outcome measures were assessed preoperatively and six months postoperatively, including the ROM, functional and clinical scores, and the radiological evaluation for retear.

The results showed significant improvements in the ROM and clinical scores within both groups postoperatively, indicating a successful surgical intervention for the LMRCTs. However, there were some differences between the groups. Group 2 showed significantly higher SSTs and UCLAs compared to Group 1. Additionally, the mean duration of surgery was shorter in Group 2, suggesting potential advantages of the PGA technique in terms of operative time.

When assessing radiological outcomes, the retear rate was slightly higher in Group 2 compared to Group 1, although the difference was not statistically significant. This indicates that both techniques are effective in reducing retear rates, but PGA may have a slightly higher risk.

LMRCTs can be treated with partial or complete repair only or tendon transfer [17]. Shoulder arthroplasty, especially reverse total shoulder arthroplasty (RTSA), is an option as it has been shown to yield better results concerning pain relief and function than hemiarthroplasty [18]. However, in some studies that evaluated patients who underwent a partial or complete repair [19] or a tendon transfer [20], they experienced less pain, but not increased muscle strength. In addition, several studies have reported a high complication rate after RTSA. According to Werner et al. [21], this was reported to be as high as 50%, and revisional surgery was performed in 33% of cases. Favard et al. [22] also reported deterioration both clinically and radiologically over time; therefore, caution is recommended when indications for RTSA are present, especially in younger patients.

As previously mentioned, it is important to repair RCTs firmly and re-establish their original footprints. However, LMRCTs are difficult to repair without residual defects, which can eventually lead to poor outcomes such as retear [23] and the superior shifting of the humeral head, which leads to the patient having to undergo shoulder arthroplasty. [24,25]. Failed rotator cuff repairs led to the investigation of patch augmentation materials to enhance the strength of the repair and facilitate healing, thus serving as an alternative technique to tendon transfer or arthroplasty for active patients with LMRCTs with minimal glenohumeral arthritis [26]. Several studies reported that patch-reinforced repair with an augmentation material inhibits gap formation at the tendon-bone repair site [27]. These studies revealed that PGA may promote the healing of the rotator cuff tendon because larger gaps have been associated with decreased healing [28].

Several reports [8,29] have shown that it is better not only to repair LMRCTs but also to reinforce them. Based on these studies, we developed the idea that arthroscopically repairing LMRCTs and then reinforcing them using SCR or PGA would lead to better outcomes for the patients. Furthermore, we attempted to determine which patch-reinforced repair option for the arthroscopic repair of LMRCTs would be more favorable for surgeons by comparing the clinical and radiological outcomes.

SCR has been suggested as an alternative treatment option for managing LMRCTs, defined as the inability of the torn rotator cuff tendon to be positioned at the original footprint [26]. The superior capsule originally attaches to a large portion of the greater tuberosity [30]. According to Ishihara et al. [31], superior capsular defects could lead to glenohumeral translation in all directions, especially superior translation at 5° and 30° of abduction. Owing to its anatomical characteristics, the reconstruction of the superior capsule can restore this superior translation of the humeral head to its prior physiological conditions [3].

Although the SCR technique is a relatively new surgical technique that is gaining popularity for its biomechanical efficacy and excellent early clinical results [17], PGA remains another valid option because it is easier for surgeons to perform than SCR. Initially, surgeons primarily used xenografts. However, according to several studies, using xenografts for PGA has shown unintended consequences [7,25,32]. There have also been several studies on PGA using synthetic patches [6,33], but they have no regenerative stimuli corresponding to the healing process. Based on these studies, we performed PGA using allogeneic dermal patches.

This study has several limitations. First, there was a risk of bias owing to the retrospective nature of the study. Second, the surgical technique was chosen by the patients. To minimize our influence on patients and the difference in their intelligence, we tried to describe the whole surgical procedure, the pros and cons, and the complications for both SCR and PGA techniques in a written document. Despite our efforts to explain the procedures to the two groups, this remains a factor that could lead to selection bias. Third, a considerable number of patients were excluded from the analysis based on the aforementioned exclusion criteria. This could have also led to selection bias in the assessment of various outcomes. Fourth, this study was based on short-term results. We evaluated the postoperative ROM angles and clinical scores on the same day that the patients visited the outpatient clinic to undergo postoperative MRI six months after the operation. Although we performed a follow-up MRI scan six months after the operation, according to Iannotti et al. [12], the time point for evaluating postoperative ROM assessments and clinical scores to predict the outcomes of the operation is still unclear. Fourth, the measurements of the ROM assessments, clinical scores, and MRI findings might be inaccurate. To reduce bias and errors, the clinical researcher was blinded to the study. MRI was also performed by several physicians. Finally, the allogeneic dermal patches used for SCR and PGA were obtained from different companies: BellaCell HD™ (Hans BioMed Corp., Seoul, Republic of Korea), SureDerm^®^ (Hans BioMed Corp., Seoul, Republic of Korea), and MegaDerm^®^ (L&C BIO, Seongnam, Republic of Korea). The heterogeneity of the allogeneic dermal patches may have also influenced the results of this study.

## 5. Conclusions

This single-center retrospective study aimed to compare the clinical and radiological outcomes of SCR and PGA as reinforcement techniques for the arthroscopic repair of LMRCTs. The study provides valuable insights into the effectiveness of SCR and PGA as reinforcement techniques for the arthroscopic repair of LMRCTs. Both techniques led to significant improvements in ROM and clinical scores, with PGA demonstrating potential advantages in terms of its operative time and functional outcomes. Therefore, this implies that SCR is a more demanding technique that requires additional surgical procedures, and that he PGA technique is a reinforcement option that is comparable to the SCR technique performed using ARCR. However, further research with larger sample sizes and longer follow-up periods is needed to confirm these findings and determine the optimal technique for LMRCT repair.

## Figures and Tables

**Table 1:** Patient demographics *.

Variables	Group 1 (*n* = 26)	Group 2 (*n* = 24)	*p* Value
Age (years)	68.96 ± 7.55	65.50 ± 7.69	0.117
Sex (Male/Female)	13/13	10/14	0.555
Side of involvement (Dominant/Non-dominant)	22/4	18/6	0.396
Duration of symptom (months)	13.56 ± 25.31	14.63 ± 26.50	0.696
Smoking (Yes/No)	3/23	3/21	0.917
Traumatic event (Yes/No)	13/13	10/14	0.555
Same-side operation history (Yes/No)	2/24	3/21	0.571
Duration of operation (minutes)	90.38 ± 20.09	83.13 ± 15.17	0.021 ^†^
Concomitant procedures			0.739
Only acromioplasty (*n*)	10 (38.5%)	4 (16.7%)	
Acromioplasty and bicep tenotomy (*n*)	12 (46.1%)	14 (58.3%)	
Acromioplasty and bicep tenodesis (*n*)	4 (15.4%)	6 (25.0%)	

* Data are presented as mean ± standard deviation; ^†^ Statistically significant.

**Table 2:** Comparison of the ranges of motion between the two groups *^,†^.

Factors	Group 1 (*n* = 26)	Group 2 (*n* = 24)	*p* Value
**Forward elevation**			
Pre-operation (°)	106.54 ± 60.92	129.17 ± 67.49	0.112
Post-operation ^‡^ (°)	157.31 ± 28.36	168.75 ± 19.18	0.099
**Internal rotation**			
Pre-operation (point)	2.50 ± 3.16	4.13 ± 3.43	0.024 ^§^
Post-operation (point)	5.92 ± 4.91	6.71 ± 4.54	0.518
**IR level difference (point)**	3.42 ± 5.17	2.58 ± 4.31	0.625

IR, internal rotation; * Data are presented as mean ± standard deviation; ^†^ All range of motion evaluations were measured when the patient actively moved; ^‡^ This was measured at six months after surgery; ^§^ Statistically significant.

**Table 3:** Comparison of functional and clinical satisfaction between the two groups *.

Factors	Group 1 (*n* = 26)	Group 2 (*n* = 24)	*p* Value
**P-VAS**			
Pre-operation	6.46 ± 1.65	6.08 ± 2.43	0.776
Post-operation ^†^	2.58 ± 1.30	2.63 ± 1.31	0.904
**SST**			
Pre-operation	3.85 ± 2.49	4.08 ± 2.10	0.543
Post-operation	6.73 ± 2.14	8.08 ± 2.14	0.034 ^‡^
**UCLA**			
Pre-operation	13.12 ± 4.61	11.38 ± 4.22	0.114
Post-operation	23.15 ± 5.38	32.50 ± 10.88	0.001 ^‡^
**ASES**			
Pre-operation	44.43 ± 16.93	44.24 ± 16.84	0.816
Post-operation	74.50 ± 9.98	71.61 ± 12.47	0.448
**CSS**			
Pre-operation	38.12 ± 18.85	36.00 ± 15.61	0.778
Post-operation	48.46 ± 14.69	54.50 ± 10.56	0.150

P-VAS, pain visual analog scale; SST, Simple Shoulder Test score; UCLA, University of California in Los Angeles shoulder score; ASES, American Shoulder and Elbow Surgeons shoulder score; CSS, Constant Shoulder Score; * Data are presented as mean ± standard deviation; ^†^ This was measured at 6 months after surgery; ^‡^ Statistically significant.

**Table 4:** Comparison of retears using postoperative MRI between the two groups *.

Retear	Group 1 (*n* = 26)	Group 2 (*n* = 24)	*p* Value
No	18 (69.2%)	16 (66.7%)	0.846
Yes	8 (30.8%)	8 (33.3%)	0.590
Partial-thickness	6	5	
Full-thickness	2	3	

MRI, magnetic resonance imaging; * This was evaluated six months after surgery.

## Data Availability

The data are not publicly available due to there being no appropriate site for uploading them at present. The data presented in this study are available on request from the corresponding author.
